# A systematic review of dietary interventions for gestational weight gain and gestational diabetes in overweight and obese pregnant women

**DOI:** 10.1007/s00394-017-1567-z

**Published:** 2017-11-11

**Authors:** Reeta Lamminpää, Katri Vehviläinen-Julkunen, Ursula Schwab

**Affiliations:** 1 Department of Nursing Science, Faculty of Health Sciences, University of Eastern Finland, P.O. BOX 1627, 70211 Kuopio, Finland; 2 Department of Nursing Science, Faculty of Health Sciences, University of Eastern Finland and Kuopio University Hospital, 70029 Kuopio, Finland; 3 Institute of Public Health and Clinical Nutrition, Faculty of Health Sciences, University of Eastern Finland, 70211 Kuopio, Finland; 4 Institute of Clinical Medicine, Internal Medicine, Kuopio University Hospital, 70029 Kuopio, Finland

**Keywords:** Dietary intervention, Gestational weight gain, Gestational diabetes, Systematic review

## Abstract

**Purpose:**

The number of overweight and obese women is increasing in the obstetric population. The aim of this study was to review studies that reported results related to the efficacy of dietary interventions on gestational weight gain (GWG) or the prevention of gestational diabetes (GDM) in overweight and obese women.

**Methods:**

The search was performed using the CINAHL, PubMed, Scopus and Medic electronic databases and limited to the years between 2000 and March 2016. This systematic review includes 15 research articles of which 12 were randomized controlled trials, and three were controlled trials. Three main categories emerged as follows: (1) the types of interventions, (2) the contents of the interventions and (3) the efficacy of the intervention on GWG and the prevention of GDM. The quality of the selected studies was evaluated using the AHRQ Methods Reference Guide for Effectiveness and Comparative Effectiveness Reviews.

**Results:**

Of the selected 15 studies, eight included a specified diet with limited amounts of nutrients or energy, and the others included a dietary component along with other components. Ten studies reported significant differences in the measured outcomes regarding GWG or the prevention of GDM between the intervention and the control groups.

**Conclusions:**

This review confirms the variability in the strategies used to deliver dietary interventions in studies aiming to limit GWG and prevent GDM in overweight and obese women. Inconsistency in the provider as well as the content of the dietary interventions leaves the difficulty of summarizing the components of effective dietary interventions.

##  Introduction

Obesity is a public health concern in Finland and other developed countries. Overweight is defined as a body mass index (BMI) ≥ 25, and obesity is defined as a BMI ≥ 30 [1]. Generally, in European countries, the percentages of obese, pregnant women vary from 7%, in Poland, to 21%, in Scotland. In Norway, Sweden, Malta and Denmark, the percentages were 12% in 2010, which were quite similar to those in Finland [2]. In 2015, in Finland, the average pre-pregnancy BMI of women giving birth was 24.6 kg/m^2^, and every third woman giving birth was categorized as overweight, while 13% were categorized as obese [3].

Overweight and obesity can lead to significant medical problems during pregnancy [4]. These conditions are associated with numerous maternal, foetal and neonatal complications [5, 6]. Overweight and obese women have an increased risk of gestational hypertension, preeclampsia and gestational diabetes (GDM) [4, 7, 8]. For the foetus, the risks caused by maternal overweight and obesity include childhood obesity, insulin resistance, and hyperlipidaemia [9]. Excessive weight gain during pregnancy is also associated with a greater newborn birthweight and a risk of childhood overweight and obesity [10].

GDM is defined as an abnormal glucose tolerance that develops or is recognized in pregnancy. The definition includes those women with previously undiagnosed diabetes and those with pregnancy-induced high glucose levels. The diagnosis is based on glucose levels in a 75 g oral glucose tolerance test (OGTT). In Finland, the proportion of women with GDM has increased rapidly during the past years from 10% in 2008 to 16% in 2015 [3].

For overweight and obese women, the recommended weight gain is less than that for normal weight women, which is 7–11.5 kg for those with a BMI of 25–29.9 and only 5–9 kg for those with a BMI of 30 or above [11]. It has been shown that women who can limit their gestational weight gain (GWG) are also more likely to show less weight retention after pregnancy [12]. Ideally, obese women should be advised and motivated to maintain a healthy diet, exercise and manage their weight even at pre-conception and throughout the pregnancy [13]. Because overweight and obesity are associated with numerous risks during pregnancy, it would be ideal to prevent the risks by losing weight pre-conceptionally. However, this is difficult to achieve; thus, lifestyle interventions are needed [9].

Reviews related to the impact of different interventions on overweight and obese pregnant women have been conducted [14–20]. A perspective of these previous reviews shows the wide range of the effectiveness of the interventions on GWG, energy intake during pregnancy, pregnancy outcomes and prevention of GDM. The studies included in these reviews also range from dietary interventions to lifestyle and exercise interventions. The results of these studies on overweight and obese pregnant women reflect the variability in the impact of the interventions and the quality of the studies, and, therefore, implications for future studies or practice are not consistent. A systematic review by Flynn and colleagues [21] focused on the effective approaches in intervention studies (*n* = 13) that aimed to reduce obesity in pregnancy. The results confirmed the difficulty of developing clinical guidelines based on the large methodological variability in the previous studies. However, there appears to be evidence that diet-based interventions are effective in controlling GWG and reducing the adverse pregnancy outcomes as compared with other methods [15, 21, 22]. A meta-analysis by Rogozinska et al. (2016) managed to identify the components, including GDM, of maternal composite outcomes that are required for assessment of the diet and lifestyle interventions in pregnant women [23]. To the best of our knowledge, only a few reviews have focused on interventions targeted for overweight and obese pregnant women aiming to prevent GDM and do not report results focused on specifically the role of dietary interventions but include other interventions as well, which makes it difficult to conclude the effect of dietary components. In this review, the interventions are limited with dietary interventions or interventions including a dietary component to explore the efficacy of diet-based interventions on overweight and obese pregnant women.

It is important to identify the effective dietary components in the interventions that are effective in GWG or the prevention of GDM in an overweight and obese pregnant population. The aim of this systematic literature review was to answer the following research question: “What are the strengths and weaknesses of dietary intervention studies focusing on GWG and the prevention of GDM?” The aim was to describe the efficacy of dietary interventions—targeted for overweight and obese pregnant women—that limit GWG and prevent GDM. The PICOS criteria for the inclusion of studies included population, i.e., overweight (BMI ≥ 25) or obese (BMI ≥ 30) pregnant women; intervention, i.e., whether a dietary intervention or a dietary component was included; comparisons, i.e., no intervention or intervention with a differing content; outcomes, i.e., GWG and/or GDM; and study design, i.e., randomized controlled trials or controlled trials.

## Methods

### Literature search

The literature search was performed electronically between February and March 2016 (the date of the last search was 11/3/2016). The selected electronic databases were CINAHL, PubMed, Scopus and Medic. The following search strategy was used (example in CINAHL): gestational OR pregnan* AND “weight gain” OR diabetes AND overweight OR obes* AND intervent*. The search terms were formulated with the help of a university librarian. The search was limited to publications between the years 2000 and 2016, and the results were limited to “Peer-Reviewed” and “English language”.

### Inclusion and exclusion criteria

The inclusion and exclusion criteria are shown in Table 1.

**Table 1:** The inclusion and exclusion criteria

Study inclusion criteria	Study exclusion criteria
Studies with overweight or obese pregnant women as a target group	Studies with an inadequate description of the dietary component used in the intervention
Studies with a dietary intervention or an intervention including a dietary component	Studies with only an exercise-based intervention without any dietary component
Studies with outcomes including GWG or GDM	Studies mainly focused on measuring GWG in the postpartum period
Studies written in English	Review articles, pilot studies
Studies with a randomized controlled trial or controlled trial design	Studies with small sample sizes, *n* < 40

### Search results and data evaluation

**Fig. 1:**
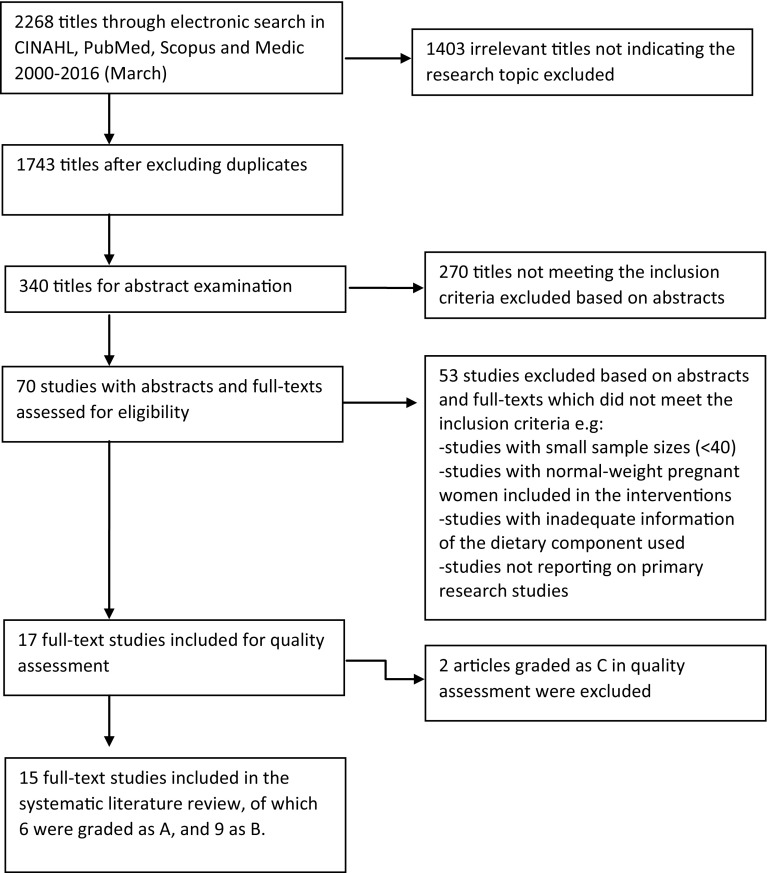
Flowchart presenting the main phases of the literature search process

First, the electronic search resulted in a total of 2268 titles, of which 1743 were identified after excluding duplicates. Altogether, 1743 titles were screened, and irrelevant titles were excluded, leaving 340 titles for careful screening based on the abstracts. The screening of the abstracts resulted in 70 remaining research articles that were subjected to a full-text examination. Of these 70 articles, 53 were excluded because they did not meet the inclusion criteria. In total, 17 articles were included in the quality assessment (Fig. 1) The quality assessment was performed using the AHRQ Methods Reference Guide for Effectiveness and Comparative Effectiveness Reviews quality assessment tool [24]. The quality assessment was performed with the cooperation of three researchers. Based on the quality assessment, six studies were graded as A; nine were graded as B; and two were graded as C. Those studies that were graded as C were excluded from the review to reduce the risk of bias at both the study and outcome levels.

Figure 1.

### Analysis of the data

The synthesis in this review was performed by analyzing the 15 selected research articles regarding dietary interventions focused on GWG and the prevention of GDM in overweight and obese women. After tabulating the studies, the results were synthesized using the content analysis method [25].

## Results

### Study characteristics

Of the 15 studies selected for this systematic review, 12 were randomized controlled trial (RCT) studies, and 3 were controlled trial (CT) studies.

The impact of the intervention was assessed on both the GDM and GWG in 4 studies [26–29], the GDM in 1 study [30], and the GWG in 10 studies [12, 31–39]. The majority of the studies were conducted in Australia (20% *n* = 3), USA (20% *n* = 3) and Denmark (20% *n* = 3). The sample sizes had a wide range from 50 to 1555. Mostly, the overweight and obese pregnant women were recruited to the intervention before the 20th gestational week (*n* = 10 studies) or during the 1st trimester of pregnancy (*N* = 5 studies). Six studies [30–34] used food records/diary as the dietary assessment method; two studies used a Food Frequency Questionnaire (FFQ) [28, 29]; and two studies used 24-hour recall [30, 37]. Two studies [12, 27] used logs for the women to report their food consumption, and an audit of items consumed was used in one study [26]. Detailed information regarding the study characteristics is shown in Table 2.

**Table 2:** Characteristics of the studies included in the review (only the outcome measures or results related to gestational diabetes mellitus (GDM) or gestational weight gain (GWG) reported in the table)

Reference details, first author, year, country	Study design	Population, subject characteristics	Outcome measures	Dietary assessment method	No. of subjects analyzed	Intervention	Results	Study quality
Markovic, T. et al. 2016, Australia	RCT	139 pregnant women at risk for GDM with an otherwise healthy single pregnancy at 12–20 gestational weeks 24 h recalls at visits 2, 3, 4	GDM	3-day food record at baseline and 34–36 weeks of gestation	125	Dietary intervention. Low glycemic index diet with protein (15–25% total energy intake E), fat 25–30E%, carbohydrate 40–45E%. One group for a low-GI diet (target GI ≤ 50) and another group for a high-fiber, moderate GI diet (HF). Five individual dietary consultations with a dietitian at 14–20,18–24, 22–28, 26–32 and 34–36 weeks of gestation	No statistically significant differences	A
Poston L. et al. 2015, UK	RCT	1555 obese pregnant women at 15–18 gestational weeks	GDM, GWG	Food frequency questionnaire at one month before randomization and for the month before the visit at 27–28 + 6 weeks of gestation	1280	Behavioral intervention. Two groups: behavioral intervention or standard care. Intervention consisted of 8 health trainer-led group or individual sessions once a week for 8 weeks. Tailored recommendations for healthy eating and advice on physical activity	No statistically significant differences	A
Wolff, S. et al. 2008, Denmark	RCT	50 non-diabetic Caucasian obese pregnant women at 15 gestational weeks	GWG	7-day food record at inclusion, 27 and 36 weeks of gestation	50	Dietary intervention. Instruction for the healthy diet according to the official Danish dietary recommendations (fat intake max.30E%, protein intake 15–20E%, carbohydrate intake 50–55E%). Energy intake was restricted and estimated individually. 10 group consultations with 1 h each with a trained dietitian for the intervention group. No consultations or restrictions on diet for control group	The women in the intervention group successfully limited their energy intake and restricted GWG measured at 36 weeks of gestation (6.6 kg vs. 13.3 kg in the controls)	A
Bogaerts, AFL. et al. 2013, Belgium	RCT	205 obese pregnant women before 15 gestational weeks	GWG	7-day food diary at baseline	197	Lifestyle intervention. The diet based on the National Dietary Recommendations consisted of 50–55% carbohydrate intake, 30–35% fat intake and 9–11% protein energy intake Three groups: - experimental group with four lifestyle intervention sessions (before 15 weeks of gestation, 18–22 weeks, 24–28 weeks and 30–34 weeks) for small groups by a midwife - a brochure group with written information on healthy lifestyle increasing physical activity -control group	GWG measured at delivery was significantly reduced in the brochure (9.5 kg) and lifestyle intervention group (10.6 kg) vs. controls (13.5 kg)	A
Petrella, E. et al.2014, Italy	RCT	61 overweight or obese pregnant women at 1st trimester of pregnancy	GWG, GDM	Food frequency questionnaire (FFQ) at baseline and at 36th gestational weeks	61	Lifestyle intervention. The diet with a macronutrient composition of 55% carbohydrate, 20% protein and 25% fat with daily calories of 1700 kcal/day (overweight women) and 1800 kcal/day (obese women). Intervention group “Therapeutic Lifestyle Changes (TLC)” led by a dietician Introduction and one-hour counseling session/follow-up at 16th, 20th, 28th and 36th week. Advice for mild physical activity included Control group received a simple booklet about lifestyle and guidelines for a healthy eating	In TLC GWG at delivery was lower vs. controls significantly in obese women but not in overweight women. The incidence of GDM was significantly lower in TLC (23.3%) comparing to controls (57.1%)	A
Vesco, K. et al. 2014, USA	RCT	118 obese pregnant women at 7–21 gestational weeks	GWG	Five-day food diary at baseline	114	Dietary intervention. Diet with an energy reduced eating plan based on the DASH dietary pattern with individualized calorie goals calculated. Women were encouraged for physical activity. Two individual counseling sessions by a dietitian and later weekly group sessions by the intervention team. Control group received one time dietary advice by a dietician	Intervention resulted in lower GWG measured at 34 weeks of gestation (5.0 kg vs.8.4 kg in controls)	A
Guenlinckx, I. et al. 2010, Belgium	RCT	195 white, obese pregnant women at 1st trimester of pregnancy	GWG	7 day food records each trimester of pregnancy	122	Lifestyle intervention 3 groups nutritional advice from a brochure -brochure and lifestyle education by a nutritionist for groups at 15, 20 and 32 weeks of gestation, including recommendations on a healthy diet (9–11% of the energy from proteins, 30–35% from fat, 50–55% from carbohydrates) -control group	No statistically significant differences	B
Haby, K. et al. 2015, Sweden	CT	319 obese pregnant women at first antenatal visit	GWG	A log book to map current diet and activity status	100	Antenatal health care intervention. Intervention group: consisted of individual extra time in antenatal visit with midwife. Dietary advice by a dietitian and midwives based on the Swedish Nutritional Recommendations, prescription for physical activity and participation in food discussion groups with dietitian was offered	Women in the intervention group had significantly lower GWG at around 38 weeks of gestation (8.6 vs.12.5%) and total weight gain less than 7 kg (36 vs.16% in controls) and lower weight postnatally	B
Vinter CA et al.2011, Denmark	RCT	360 obese pregnant women at 10–14 gestational weeks	GWG	–	304	Lifestyle intervention. For the intervention groups dietary guidance and physical activity was included. Guidance was based on the official Danish recommendations given by a dietitian individually at 4 occasions: 15, 20, 28 and 35 weeks of gestation. Women were encouraged for physical activity 30–60 min. daily and they also had free full time membership in a fitness center for 6 months with closed training classes	GWG at 35 weeks of gestation was significantly lower in the intervention group (median) 5.6 kg vs.7.0 kg in the controls	B
Artal, R. et al. 2007, USA	CT	96 obese pregnant women with GDM before 33 gestational weeks	GWG	Daily diary regarding food intake	96	Lifestyle intervention -exercise and diet group (ED) -diet group (D) Eucaloric or hypocaloric diet by a dietitian and instructions by a diabetes nurse specialist for both groups, a consistent carbohydrate meal plan: 25Kcal/kg for overweight, 20Kcal/kg for obese, 15Kcal/kg for morbidly obese women. The ED group was encouraged for exercise	Weight gain (measured at each obstetric visit, the last measurement within 2 weeks to delivery) per week was significantly lower in the ED group	B
Hawkins, M. et al. 2014, USA	RCT	88 overweight or obese Hispanic pregnant women before 18 gestational weeks	GWG	24 h recall and self-monitoring logs for the women	68	Lifestyle intervention. Two groups: -intervention group with a dietary component for decreasing intake of foods high in saturated fat and increase dietary fibre as recommended by the American Dietetic Association. Led by a health educator consisting of 6-monthly individual sessions and five telephone sessions. Women were encouraged for physical activity -standard care group	No statistically significant differences	B
Harrison, C. et al. 2013, Australia	RCT	228 overweight or obese pregnant women at risk for GDM at 12–15 gestational weeks	GDM, GWG	–	203	Lifestyle intervention consisting of four sessions individually by a health coach at 14–16, 20, 24 and 28 weeks of gestation. The intervention included pregnancy-specific dietary advice in addition to simple healthy eating and physical activity Control group received written health information	Overweight women in the intervention group gained significantly less weight in early pregnancy than in the control group in 26–28 gestational weeks, but with obese women the GWG in early pregnancy was similar. GDM prevalence 22%, less cases in the intervention group (*P* = 0.1)	B
Quinlivan, J. et al. 2011, Australia	RCT	124 overweight or obese pregnant women at first antenatal visit	GDM, GWG	Audit of items consumed in the day before the 1st and final antenatal visit	124	Four-step multidisciplinary antenatal care group including one dietary-related phase: five minute individual intervention at every antenatal visit by a food technologist asking about the women’s eating habits of the previous day and providing information on reading food labels and recipes for a healthy pregnancy diet. Standard care group with routine antenatal care	Intervention resulted in significant reduction in the incidence of GDM (6% vs.29% in controls) and reduced GWG in the last antenatal visit (7.0 kg vs.13.8 kg in controls)	B
McGiveron, A. et al. 2015, UK	CT	178 pregnant women with BMI > 35 at 16–18 gestational weeks	GWG	–	178	A midwife-led intervention. Intervention consisted of 8 sessions from around 16 weeks of gestation at every 2–4 weeks until 36 weeks of gestation. Intervention included health education around diet and exercise, accompanied by one-to-one guidance by a midwife or a healthy lifestyle advisor, and monitoring of dietary change. Non-intervention group of women who did not want to participate	In the intervention group the mean weight gain (4.5 kg) at 36 weeks of gestation was less than in the non-intervention group (10.3 kg) with association with 95% reduction in the risk of gestational hypertension	B
Renault, K. et al. 2014, Denmark	RCT	425 obese pregnant women at 11–14 gestational weeks	GWG	–	389	Dietary intervention. Two intervention groups + control: (a)physical activity and dietary intervention with a follow-up on a hypocaloric Mediterranean style diet every 2 weeks individually with a dietitian and by phone contacts (b) physical activity intervention in which women were encouraged to increase physical activity (c) controls	No statistically significant differences	B

### The types of interventions

The approach of the dietary interventions varied among the 15 selected studies. Seven studies [27, 28, 32, 34–37] referred to “Lifestyle interventions”, four [30, 31, 33, 39] studies referred to “Dietary interventions” and the remaining studies included different variations in defining the type of intervention, but all studies included a dietary component. Eight studies [28, 30–34, 36, 39] included a specified diet with limited amounts of allowed nutrients or energy in the dietary intervention. The remaining seven studies used a strategy including dietary advice or counselling related to healthy eating and a healthy diet during pregnancy mostly based on the National Nutritional Recommendations.

The counselling was provided by a nutritionist or a dietitian in most of the studies [12, 28, 30, 31, 33–36, 39], but in some cases, the counselling was provided by another health care provider, such as a health coach or a lifestyle coach, alone or accompanied by a dietitian or a nutritionist [12, 27, 29, 32, 36–38]. Midwife-led interventions were rare. The counselling was provided individually in seven studies [26, 27, 30, 35, 37–39], in groups in three studies [30, 31, 33] and both individually and in groups in three studies [12, 29, 33]. In two studies [28, 36], the mode of delivering advice was not clearly described. In addition to the individual or group sessions providing dietary advice or the counselling, some of the studies reported giving written material for the women to support the process, such as handbooks, booklets or guides, containing information related to healthy food, nutrition [29, 30, 37, 38] and benefits to avoiding excessive weight gain [38].

Exercise or physical activity was included in 10 studies [12, 27–29, 33, 35–39] as a component of the intervention. The role of physical activity varied from the mildest level of encouraging pregnant women to engage in physical activity during the intervention to interventions in which exercise and diet were combined.

### The content of the dietary interventions

The content of the diets with limited amounts of protein, fat and carbohydrate intake used in the dietary interventions are described in detail in Table 2. One study limited the energy intake to 1700 kcal/day for overweight pregnant women and 1800 kcal/day for obese pregnant women [28]. Another study used the pattern of total energy intake per day based on weight as 25 kcal/kg for overweight women and 20 kcal/kg for obese women [36]. The Dietary Approaches to Stop Hypertension (DASH) dietary pattern with individualized calculated daily calorie goals was used in one study [33], and another study referred to a Mediterranean style, hypocaloric diet [39].

### The effectiveness of the dietary interventions in relation to GWG and GDM

Of the total 15 studies, 10 studies reported significant differences between the study and the control groups in limiting the GWG (*n* = 10) and/or preventing GDM (*n* = 3), of which seven focused on GWG, and three focused on both.

The remaining five studies [29, 30, 34, 37, 39] reported no significant differences between the intervention and the control groups. The similarities among the effective interventions were a combination of a dietary component and a certain extent of physical activity. However, there were three studies [26, 31, 32] with dietary interventions or interventions with a dietary component that were effective without physical activity. The effective interventions consisted of an inclusion of a dietitian or a nutritionist, but the interventions mainly delivered by a midwife or a lifestyle/ health coach had impacts on the outcomes. The roles of midwives or nurses in the interventions were highlighted in only four studies [12, 32, 36, 38], in which each was reported as effective. Three of these studies [12, 36, 38] included physical activity, in addition to dietary advice or a specified diet.

In the five studies reporting no significant differences between the intervention and control groups, the study designs were similar to those in which the intervention was found to be effective. However, two of the non-effective interventions did not include a specified diet [29, 37], but dietary advice was included, and two studies did not include physical activity [30, 34]. There were no differences in the efficacy based on the time of recruiting the women in the intervention. One study reported that the interventions were effective with overweight women but not obese women [27], and another study reported the opposite results [28]. Other studies did not report any significant differences when participants were stratified by BMI category as overweight vs. obese women. In most of the studies, GWG was measured at the end of the pregnancy between gestational week 38 and the time of delivery [12, 26, 28, 32, 34, 36], and in three studies, GWG was measured at approximately 36 weeks of gestation [31, 38, 39]. In the remaining studies, the time of the GWG measurement varied from 26 to 35 weeks of gestation.

## Discussion

### Reflection on the results

This systematic review confirms the potential of limiting the GWG or preventing GDM through different dietary interventions in overweight or obese pregnant women since the majority of the studies reported significant differences between the study and control groups. However, this review also confirms the variability in the strategies used to deliver dietary interventions in studies aiming to limit GWG and prevent GDM and, hence, the difficulty of summarizing the components of effective dietary interventions [22]. Dietary interventions were selected for this review because they have been previously reported to be the most effective type of interventions for limiting the GWG and preventing GDM [21].

Although the majority of the selected studies reported the interventions to be effective, there still appears to be inconsistency in the results since there were five studies that reported no significant differences between the groups but consisted of similar components as those studies that reported that the interventions were effective. The time of measuring the GWG varied from as early as 26 weeks of gestation until the day of delivery, but in those two studies reporting GWG at approximately 26 weeks of gestation [27, 29], the GWG was not either the main outcome measure or titled as “GWG in early pregnancy”. It appears to be difficult to note other specific weaknesses in the studies in relation to the study design, participants or delivery of the dietary intervention.

Despite limiting this systematic review to studies including a dietary-intervention and the assessed outcome of the studies including either GWG or GDM, there still appears to be much variation in the dietary approach within the studies. Some studies focused on a specified diet with limited amounts of nutrients or energy and others used strategies of delivering dietary advice or counselling based on National Recommendations. A certain extent of physical activity was also included in most of the studies. The impact of the varying dietary approaches on GWG and GDM remain unclear because the interventions consisted of different components with additional variation in the implementation of the components, such as individual vs. group-based counselling.

In a review by Heslehurst and colleagues [40] it was stated that most of the intervention studies related to supporting weight management during pregnancy have focused on changing the pregnant women’s behaviour. These studies have not focused on encouraging public health nurses (PHN) or other health professionals, such as midwives, to implement weight management guidelines into practice and provide counselling related to these issues. These practices could be important in motivating women. The commitment to the lifestyle changes required by the interventions is often difficult to achieve [40, 41]. It has been suggested that midwives should be offered support and education to keep up to date with the guidance on weight management and behaviour change techniques during pregnancy because they play a key role in health promotion [15].

Interestingly, in this systematic review, the midwives’ or nurses’ role in the interventions was mentioned in a minority of the studies. In addition, dietary advice was less often focused on groups, which could offer plenty of advantages both at the organizational and individual level since it is more cost-effective and offers peer-support for the women, which can have a positive effect as well on compliance with the intervention.

The quality of the studies assessed by the AHRQ Methods Reference Guide for Effectiveness and Comparative Effectiveness Reviews quality assessment tool [24] was generally good, and there were no differences in the efficacy of the interventions in relation to the assessed quality of the study regardless of whether it was graded as A or B. The AHRQ quality assessment tool is tailored for evaluating studies with dietary-interventions. In this review, not all of the selected studies included an actual dietary-intervention, but a dietary component with advice or counselling without detailed information was included; thus, the quality assessment tool was not entirely applicable to all of the studies. This may have some effect on the grading results.

Most of the studies (*n* = 13) selected in this systematic review were conducted in the year 2010 and beyond. This probably reflects the fact that overweightness and obesity are increasing health concerns in the obstetric population, and there is a need to develop interventions that could impact the weight management and health of the pregnant women and their newborns.

In future research it would be crucial to tailor the duration and contents of dietary interventions more consistently, for example based on national nutritional recommendations. More importance should be placed as well on the provider of the dietary intervention to allow for both the dietitian and the midwife or the public health nurse in the antenatal care to be deeply involved in the intervention. This could enable a smooth transition of the experiences from trials to every day practice in maternal care, which could also improve the quality of antenatal dietary counselling targeted for overweight and obese pregnant women.

### Limitations of the study

A limitation of this study is the exclusion of studies written in languages other than English, which may cause a minor publication bias. The exclusion of pilot studies may have slightly reduced the number of studies included in this review; however, this exclusion is related to the quality of the review. The search did not encompass grey literature (e.g., theses, conference proceedings, etc.), which may cause some bias in the results of this review.

In the search and the selection of the articles for this review the behaviour change technique (BCT) taxonomy was not used to help in categorising the interventions or components used in the studies, which could have provided clearer results in this review [42]. Instead, each study was carefully screened to determine the type and components of the dietary intervention used.

## Financial support

This study has been financially supported by the Finnish Foundation for Nurse Education and the Finnish Cultural Foundation for RL as research grants.
